# Diagnostic Reassessment of a Historical Case of Atypical Heparin-Induced Thrombocytopenia: Between Spontaneous Heparin-Induced Thrombocytopenia and a Vaccine-Induced Immune Thrombotic Thrombocytopenia-Like Syndrome

**DOI:** 10.3390/life15111767

**Published:** 2025-11-18

**Authors:** Jordan Wimmer, Solène Kirscher, Manon Dolt, Agathe Herb, Léa Pierre, Lélia Grunebaum, Olivier Feugeas, Laurent Sattler, Dominique Desprez

**Affiliations:** 1 Laboratoire D’hématologie Biologique, Unité Hémostase, Hôpitaux Universitaires de Strasbourg, 67200 Strasbourg, France; 2 Centre de Ressource et Compétence des Maladies Hémorragiques Constitutionnelles, Hôpitaux Universitaires de Strasbourg, 67200 Strasbourg, France

**Keywords:** autoimmune heparin-induced thrombocytopenia, PF4-disorders, spontaneous heparin-induced thrombocytopenia, VITT-like syndrome, cerebral venous sinus thrombosis

## Abstract

PF4-dependent disorders encompass a heterogeneous group of immune-mediated thrombotic syndromes, including heparin-induced thrombocytopenia (HIT), its autoimmune variants such as spontaneous HIT, and vaccine-induced immune thrombotic thrombocytopenia (VITT). The recent identification of VITT and VITT-like entities has significantly expanded the diagnostic spectrum, complicating the retrospective interpretation of cases that occurred before their formal recognition. We report the case of a young patient who initially presented with a clinical and biological presentation suggestive of atypical HIT, at a time when neither spontaneous HIT nor VITT were defined. The patient was re-evaluated during the COVID-19 vaccination campaign, prompting a reassessment of the initial diagnosis in light of current knowledge on PF4-related disorders, which continue to increase in both diversity and complexity. A critical review of clinical and laboratory findings now favors a diagnosis of VITT-like syndrome over spontaneous HIT, although confirmatory testing is no longer feasible given the time elapsed since the acute phase. This case highlights the importance of revisiting historical cases using updated diagnostic criteria to improve the identification and management of these emerging and underrecognized syndromes.

## 1. Introduction

Antibodies directed against platelet factor 4 (PF4) are at the origin of two major clinical entities that are distinct but have strong clinical and biological similarities: heparin-induced thrombocytopenia (HIT) and vaccine-induced immune thrombosis and thrombocytopenia (VITT). These entities are characterized by a common pathogenic mechanism involving the formation of antibodies directed against PF4 tetrameric complexes, liable to produce a prothrombotic coagulopathy associated with thrombocytopenia. First described in 1973 [1], HIT is an immunological complication resulting from exposure to heparin, leading to the formation of pathogenic antibodies classically directed against the PF4/heparin complex. HIT is, however, a much more complex entity, comprising a wide variety of syndromes ranging from classical HIT (cHIT) to highly atypical forms such as autoimmune HIT (aHIT) and spontaneous HIT (spHIT).

The VITT syndrome, described during the COVID-19 vaccination campaign, is a rare but serious complication of adenoviral vector-based vaccines against SARS-CoV-2 (ChAdOx1 nCoV-19 and Ad26.COV2.S vaccines) [2, 3, 4]. This condition, characterized by the presence of high-affinity antibodies directed against PF4 alone, has significantly contributed to the deepening of the understanding of the pathophysiological mechanisms underlying all PF4-dependent disorders. Numerous similarities between certain past or current cases of “atypical” presentations of HIT and VITT syndrome, particularly following adenoviral infections, suggest the existence of VITT-like syndromes whose pathophysiological mechanisms are closer to VITT than to HIT [5, 6]. Thus, it can sometimes be difficult to definitively determine the diagnosis in certain patients when distinguishing between atypical forms of HIT, such as spHIT, and VITT-like entities—particularly in patients whose clinical history predates the emergence of the VITT concept, and for whom the complementary diagnostic tests now available for differential diagnosis cannot be performed retrospectively in the absence of frozen aliquots preserved from the acute phase.

We report here the case of a female patient whose clinical history we previously published in November 2010 [7], under the diagnosis of atypical-HIT, for which we are now considering a reclassification as spHIT or VITT-like syndrome in the current classification [1]. This case highlights the long-term diagnostic uncertainty that can persist in such historical cases, particularly when new medical developments require clinical reassessment. For example, during the COVID-19 vaccination campaign, clinical decision-making was complicated for patients with a past history of atypical PF4-dependent syndromes, raising concerns regarding the safety and appropriateness of vaccination in the absence of a clear diagnostic framework.

## 2. Case Presentation

In 2010, we reported the case of a 13-year-old female patient admitted to the pediatric intensive care unit for persistent headaches lasting several days, accompanied by a febrile episode, followed by altered consciousness and an epileptic seizure event.

On admission, laboratory tests revealed mild thrombocytopenia at 145 × 10^9^/L (reference: 150–450 × 10^9^/L), along with a biological inflammatory syndrome, marked by elevated C-reactive protein (CRP) at 21 mg/L (reference: <5 mg/L) and neutrophilic leukocytosis at 15 × 10^9^/L (reference: 1.8–7.9 × 10^9^/L). Brain MRI identified a cerebral venous sinus thrombosis (CVST) involving the superior sagittal sinus. The patient had no personal or family history of thrombosis, nor any prior exposure to heparin. Infectious workup revealed acute otitis media and bilateral frontal sinusitis, though no pathogen was isolated. Thrombophilia screening was unremarkable, except for a transient, mild protein S deficiency at 45% (reference: >65%), which later normalized. Testing for lupus anticoagulant was positive, while antiphospholipid antibodies were negative, including IgM/IgG anticardiolipins, anti-β2-glycoprotein I, anti-prothrombin, anti-serine, anti-phosphatidylethanolamine, and anti-annexin V antibodies.

Upon diagnosis of CVST, therapeutic anticoagulation with unfractionated heparin (UFH) was promptly initiated. Twelve hours later, there was a worsening of thrombocytopenia (platelet count dropped to 108 × 10^9^/L), without overt signs of coagulopathy, except for a slight decrease in prothrombin time to 63% (reference: >70%) (D-dimer levels were not assessed at the time). Given the lack of prior heparin exposure and a low HIT probability score (2 points, according to the 4T clinical pre-test score), anticoagulation was continued.

However, due to the persistence of thrombocytopenia and despite no progression of the thrombotic event, testing for anti-PF4/heparin IgG antibodies was performed using an enzyme-linked immunosorbent assay (ELISA), which returned strongly positive with an optical density (OD) of 2.8 (positivity threshold: 0.50), using the Asserachrom HPIA IgG kit (Diagnostica Stago, Asnières-sur-Seine, France). This unexpectedly strong positivity prompted further testing, two days later, using two additional commercial ELISA kits which were also strongly positive: OD = 2.3 (positivity threshold: 0.48) with the Hyphen Zymutest HIA IgG/A/M kit (Hyphen Biomed, Neuville-sur-Oise, France) and OD = 2.1 (positivity threshold: 0.48) with the Immucor GTI Diagnostics kit (Immucor, Waukesha, WI, USA), with OD dropping to 0.13 upon addition of heparin.

**Figure 1:**
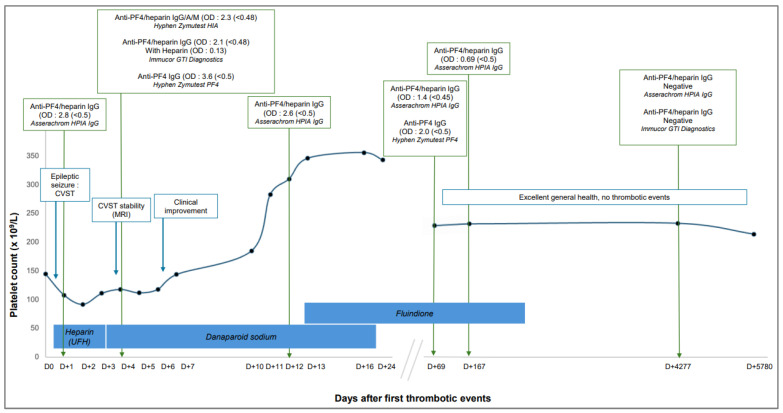
Main clinical and laboratory data of the patient during the time.

Despite these serological findings, functional platelet activation assays remained negative, including both heparin-induced platelet aggregation test and serotonin release assay (SRA). In light of this atypical clinical and biological HIT-like presentation, especially in the absence of prior heparin exposure, a dedicated ELISA for anti-PF4 IgG antibodies was performed. This test returned strongly positive, with an OD of 3.6 (positivity threshold: 0.50) using the Hyphen Zymutest PF4 kit (Hyphen Biomed, Neuville-sur-Oise, France). Given the strong positivity for anti-PF4/heparin antibodies, and despite negative functional assays, a decision was made to discontinue anticoagulation with UFH and switch to therapeutic-dose danaparoid sodium. An ELISA (Asserachrom HPIA IgG kit), performed on day 12 of hospitalization, revealed persistent strong positivity (OD = 2.6; positivity threshold: 0.50). The patient’s clinical condition gradually improved, and platelet counts normalized, reaching 346 × 10^9^/L by day 14 of hospitalization and anticoagulation was transitioned to fluindione. The clinical course continued to improve progressively, and the child was discharged after 24 days of hospitalization (The main clinical and laboratory data are summarized in Figure 1).

At three months after the episode, the neurological examination was entirely normal, and anticoagulation with fluindione was maintained for a total duration of one year. Biologically, the platelet count was normal without thrombocytopenia (219 × 10^9^/L). Autoimmune screening revealed signs of dysimmunity, with weakly positive antinuclear antibodies at a titer of 1:160, and the emergence of IgG anti-phosphatidylethanolamine antibodies at 25 IU/L (reference range: 0–10 IU/L). Testing for anti-PF4/heparin IgG antibodies (Asserachrom HPIA IgG kit) remained positive, with an OD of 1.4 (positivity threshold: 0.45), as did the test for anti-PF4 IgG antibodies alone (Hyphen Zymutest PF4 kit), with an OD of 2.0 (positivity threshold: 0.50). Tests for lupus anticoagulant were negative, confirming the transient nature of this antibody positivity, which was likely secondary to the infectious episode.

At six months, the autoimmune workup returned negative, while anti-PF4/heparin antibody testing remained slightly positive, with an OD of 0.69 (positivity threshold: 0.50) (Asserachrom HPIA IgG kit).

She was re-evaluated in 2021, 11 years after the initial episode, in the context of the emergence of the first reported cases of VITT following COVID-19 vaccination [6]. Clinically, the patient was in excellent general health. She had experienced no thrombotic events, no autoimmune manifestations, had undergone no surgical procedures, and had not been pregnant.

Regarding COVID-19, the patient reported a mild infection during the first wave of the pandemic and had received her first dose of the ChAdOx1 nCoV-19 vaccine just a few days prior to the consultation. Biologically, she showed no thrombocytopenia (233 × 10^9^/L), negative D-dimer levels (<270 µg/L), and negative testing for lupus anticoagulant. Anti-PF4/heparin IgG antibodies (Asserachrom HPIA IgG kit) and anti-PF4 IgG antibodies alone (Immucor PF4 GTI Diagnostics kit) were both negative. As a precautionary measure, and in light of her history of PF4-dependent pathology and the limited understanding of the newly emerging VITT phenomenon at that time, continuation of the adenoviral vector-based vaccination schedule was discouraged, in favor of an mRNA-based vaccine (either Spikevax or Comirnaty). It was also reiterated that heparin and its derivatives remain strictly contraindicated, given her medical history.

Finally, the patient was seen again in 2025 for a follow-up consultation, during which she remained in excellent general health, with no thrombotic or autoimmune events, and anti-PF4 antibody testing remained negative.

## 3. Discussion

This case illustrates the diagnostic complexity posed by clinico-biological presentations of PF4-related disorders, in which distinguishing between a spontaneous form of aHIT and a VITT-like syndrome remains a challenge, particularly for cases that precede the emergence of the VITT entity. The main clinical and biological features of these different entities are summarized in Table 1.

***Autoimmune HIT (aHIT):***

Beyond the classical form of HIT, which is typically associated with prior heparin exposure and manifests as an immune-mediated thrombotic thrombocytopenic syndrome due to the formation of anti-PF4/heparin antibodies, there exist historically termed “atypical forms” that are now collectively classified as aHIT.

The aHIT group comprises a subset of entities that share close clinical and biological similarities with classical HIT, but whose pathophysiology involves heparin-independent platelet activation [8]. Certain entities in this group are triggered within 5–10 days of heparin exposure, but may also occur after discontinuation:

- Delayed-onset HIT, characterized by a HIT-like syndrome combining thrombocytopenia and frequent thrombotic events often occurring in unusual sites, which appear or worsen after discontinuation of heparin therapy [9, 10, 11].
- Refractory HIT, defined by the persistence of thrombocytopenia for several weeks after heparin withdrawal, and appears to be associated with the continued presence of antibodies capable of heparin-independent platelet activation [11, 12]. This form typically resolves following intravenous administration of polyvalent immunoglobulins [13, 14].
- Flush-heparin HIT which refers to a presentation occurring after exposure to very small amounts of heparin, and also leads to the development of antibodies with heparin-independent platelet-activating properties [15, 16].
- Fondaparinux-associated HIT which refers to cases occurring after exposure to fondaparinux, in which cross-reactivity of antibodies with this anticoagulant is demonstrated in vitro, along with increased positivity of functional platelet assays in the presence of fondaparinux [17, 18].

In contrast to cHIT, which is usually characterized by a predominance of venous thromboses in large-caliber vessels and moderate thrombocytopenia (50–100 × 10^9^/L), aHIT is distinguished by a higher frequency of venous thromboses in small-caliber or atypical sites (such as cerebral or splanchnic veins), a pattern that is even more pronounced in spHIT, and by more severe thrombocytopenia (often <50 × 10^9^/L) [19]. In response to the thrombotic situation, heparin therapy is sometimes initiated, which may in fact worsen the thrombocytopenia [12, 20].

Spontaneous HIT is the aHIT entity that occurs without prior heparin exposure but within 5 to 10 days following exposure to a presumed triggering factor, arising mainly from two clinical situations:

- A recent history of orthopedic surgery, especially knee arthroplasty, in which patients develop a clinical HIT-like syndrome sometimes even while receiving alternative, non-heparin anticoagulation, such as vitamin K antagonists [21, 22]. These cases frequently exhibit a strong female predominance, a high rate of thrombotic events, and often hemorrhagic adrenal gland necrosis [19].
- A recent infectious syndrome (such respiratory, skin or oral infections, of either bacterial or viral origin), with patients typically presenting with thrombosis and thrombocytopenia, or with a rapid decline in platelet count following heparin administration. The thrombotic profile in these situations is generally marked by venous thromboses, particularly CVST [19].

The common feature across all cases of spHIT is the strong positivity for antibodies targeting the PF4 or the PF4/heparin complex, as detected by ELISA, using samples collected prior to any heparin exposure. It is suggested that other polyanionic substances, released either in the context of orthopedic surgery or an infectious syndrome, may play a pathophysiological role in triggering the formation of these antibodies. Indeed, several non-heparin polyanions have been shown to substitute for heparin in facilitating antibody formation, including pentosan polysulfate [23], chondroitin sulfate [24], nucleic acids [25], and bacterial membrane proteins [26, 27, 28]. Additionally, there is a high frequency of positive results in functional platelet activation assays that are heparin-independent [19]. Unlike cHIT IgG antibodies, which are polyclonal, exhibit low avidity for the neoepitope on the polar region of PF4 generated by PF4-heparin binding, and whose immune complexes trigger heparin-dependent platelet activation, aHIT IgG antibodies are also polyclonal but display higher avidity for the same polar binding site on PF4, even in absence of heparin, with their immune complexes inducing heparin-independent platelet activation [8].

***VITT and VITT-like syndromes:***

VITT is a clinico-biological entity that emerged in early 2021, with several case series reporting the occurrence of a syndrome characterized by an high rate of venous thromboses in small-caliber vessels and unusual sites (predominantly cerebral and sometimes splanchnic) associated with often severe thrombocytopenia and laboratory evidence of consumptive coagulopathy (elevated D-dimer levels), typically occurring within 5 to 30 days following administration of a first dose of an adenoviral vector-based SARS-CoV-2 vaccine (ChAdOx1 nCoV-19 and Ad26.COV2.S) [2, 3, 4, 29]. The estimated incidence is 1–2 cases per 100,000 vaccinations [3, 29], and the mortality rate is very high, exceeding 50% in some series, with rapidly deteriorating clinical courses, including cerebral hemorrhagic transformation, intracranial hypertension and extensive thrombosis. Several reports have also described worsening of thrombotic phenomena following platelet transfusion [2, 3].

From a pathophysiological perspective, this entity results from the formation of IgG -antibodies, without prior exposure to heparin, specifically directed with a high avidity against an equatorial epitope of PF4 that overlaps with the heparin-binding site [30, 31]. These immune complexes, similarly to those in HIT, are capable of triggering a thromboinflammatory process through an immunothrombotic mechanism [32, 33], due to their heparin-independent platelet-activating properties, mediated via the FcγIIa receptor [4]. Furthermore, VITT antibodies are monoclonal or oligoclonal, unlike the polyclonal antibodies observed in HIT [34], suggesting a potential genetic predisposition to developing VITT [35].

Following the emergence of VITT and the growing understanding of its pathophysiological mechanisms, striking similarities with several earlier cases described before 2021, some of which were historically classified as aHIT [36], have led to the description of a VITT-like syndrome. This syndrome shares numerous clinical features (such CVST and hemorrhagic transformation) and biological findings (including sometimes profound thrombocytopenia and the presence of anti-PF4 antibodies with heparin-independent platelet activation properties) with VITT, but occurs in the absence of prior heparin exposure or recent adenoviral vector vaccination [36]. The antibodies detected in these VITT-like syndromes exhibit very similar characteristics to those identified in VITT induced by adenoviral vector vaccines [37].

Several cases have been reported following recent vaccination against human papillomavirus [38] or SARS-CoV-2 using mRNA technology [39]. Similarly, a few cases have occurred after infectious syndromes caused by adenoviruses [5, 40] as well as other viruses such as respiratory syncytial virus (RSV) or cytomegalovirus (CMV) [36, 41, 42], or of unknown etiology [6, 40], suggesting that the triggering factors are likely diverse and multiple, and that the incidence of this syndrome is probably underestimated. Management is also similar to that of VITT syndromes, involving curative anticoagulation (often non-heparin-based) and inhibition of platelet activation mediated by the FcγIIa receptor, such as with intravenous polyvalent immunoglobulins or corticosteroid therapy [25].

**Table 1:** Comparative table of the main characteristics of PF4-dependant diseases; references [1, 2, 3, 4, 8, 19, 29, 43, 44, 45, 46]. **Abbreviations**: Adenoviral vector vaccine (AAV); Bilateral adrenal hemorrhage (BAH); Cerebral venous thrombosis (CVT); Heparin-induced platelet activation assay (HIPA); Mesenteric venous thrombosis (MVT); PF4-induced platelet activation assay (PIPA); Rapid immunoassays (RIA); Serotonin-release assay (SRA); Unusual site thrombosis (UST).

	Classic HIT	Auto-Immune HIT	Spontaneous HIT	VITT	VITT-Like Syndrome
**Trigger**	Heparin	Heparin	No proximate heparin Knee surgery or Infectious syndrome	No proximate heparin COVID-19 AAV	No proximate heparin or COVID-19 AAV Infectious syndrome, non-AAV, other trigger?
**Onset time**	5–10 days after starting heparin	5–10 days after immunizing exposure to heparin (sometimes persists after stopping)	5–10 days after the triggering events occur	5–30 days after COVID-19 AAV administration	A few days to a few weeks after the triggering events occur
**Antibody type**	Low-avidity, polyclonal anti-PF4/heparin IgG	High-avidity, polyclonal anti-PF4 IgG AND Low-avidity, polyclonal anti-PF4/heparin IgG	High-avidity, polyclonal anti-PF4 IgG	High-avidity, mono-oligoclonal anti-PF4 IgG	High-avidity, mono-oligoclonal anti-PF4 IgG
**Thrombocytopenia**	Often mild to moderate (50–100 G/L)	Often severe (<50 G/L)	Often severe (<50 G/L)	Often severe (<50 G/L)	Often severe (<50 G/L)
**Thrombotic events**	Usually macrovascular (venous > arterial) thrombosis Low frequency of UST	Usually macrovascular (venous > arterial) thrombosis Higher frequency of UST	Usually macrovascular (venous > arterial) thrombosis Higher frequency of UST (CVT, MVT, BAH)	High frequency of UST (CVT, MVT) sometimes followed by hemorrhagic transformation	High frequency of UST (CVT, MVT) sometimes followed by hemorrhagic transformation
**PF4-dependant ELISA**	Solid-phase: High sensitivity for both anti-PF4/heparin and anti-PF4 tests Fluid-phase: Anti-PF4/heparin > Anti-PF4	Solid-phase: High sensitivity for both anti-PF4/heparin and anti-PF4 tests Fluid-phase: Anti-PF4/heparin > Anti-PF4	Solid-phase: High sensitivity for both anti-PF4/heparin and anti-PF4 tests Fluid-phase: Anti-PF4/heparin > Anti-PF4 test	Solid-phase: High sensitivity for both anti-PF4/heparin and anti-PF4 tests Fluid-phase: Anti-PF4 > Anti-PF4/heparin	Solid-phase: High sensitivity for both anti-PF4/heparin and anti-PF4 tests Fluid-phase: Anti-PF4 > Anti-PF4/heparin
**PF4-dependant RIA**	High-sensitivity	High-sensitivity	High to moderate-sensitivity	Low-sensitivity	Low-sensitivity
**Platelet activation test**	Heparin-dependent activation SRA: Higher sensitivity with heparin and/or PF4 HIPA/PIPA: High sensitivity	Heparin-independent activation SRA: Higher sensitivity with heparin and/or PF4 HIPA/PIPA: High sensitivity	Heparin-independent activation SRA: Higher sensitivity with PF4 > Heparin HIPA/PIPA: High sensitivity	Heparin-independent activation SRA: Higher sensitivity with PF4 Inhibition in presence of heparin PIPA > HIPA	Heparin-independent activation SRA: Higher sensitivity with PF4 Inhibition in presence of heparin PIPA > HIPA

***About the case: spHIT or VITT-like syndrome?***

The emergence of new knowledge regarding the classification and diagnosis of PF4-disorders, which was unavailable at the time of our patient’s case, prompts us to reconsider its diagnostic reclassification.

The absence of prior exposure to heparin, as well as to COVID-19 vaccination, rules out both classical HIT and post-vaccination VITT, and instead supports consideration of diagnostic entities potentially triggered by alternative factors. Moreover, the clinical presentation resembles that typically observed in spHIT [19] and VITT-like syndromes [6, 36, 38, 39, 40, 41, 42], with a CVST preceded by severe headaches in an infectious context, occurring within a timeframe compatible with these entities, assuming the infection acted as a trigger. Although the initial thrombocytopenia was less severe than is usually reported in such conditions, several cases of spHIT or VITT-like syndromes have been documented with only mild initial thrombocytopenia [5, 19]. In light of these clinical features, a distinction between these two entities cannot be reliably established.

Laboratory testing constitutes a cornerstone in the diagnosis of PF4-disorders, encompassing serological detection of HIT- or VITT-like antibodies, alongside functional assays that confirm their pathogenic platelet-activating properties. Antibody detection techniques based on the immunoenzymatic ELISA principle demonstrate excellent sensitivity for spHIT and VITT IgG antibodies, whether using solid-phase kits with PF4/heparin, PF4/polyvinylsulfonate, or PF4 alone, and frequently yield very strong positivity for spHIT and VITT [25, 43]. This was likewise observed in our case, with strong positivity obtained across three different solid-phase kits. Therefore, it appears difficult to distinguish HIT from VITT using these ELISA tests. However, it has been shown that adding heparin to these ELISA tests leads to a negative result in cases of VITT, unlike HIT, probably due to competition for the common PF4 binding site between VITT antibodies and heparin [31]. Consequently, the disappearance of the ELISA signal after the addition of heparin provides preliminary support for a diagnosis of a VITT-like syndrome rather than spHIT.

Furthermore, a study evaluating the performance of an ELISA technique using PF4 or a PF4/heparin mixture in the fluid phase, in relation to these different entities, demonstrated better specificity for classic HIT (better positivity with the PF4/heparin test compared to PF4 alone) and for VITT (negation of the fluid-phase ELISA with PF4/heparin and strong positivity maintained with PF4 alone) [44]. Regarding aHIT, including spHIT, the results are not sufficiently conclusive, although they suggest better specificity of the anti-PF4/heparin tests compared to PF4 alone. This fluid-phase ELISA technique would at least allow for improved diagnosis of VITT when there is a negative PF4/heparin profile and a positive PF4 alone profile. Such a test would have been of considerable interest at the time of the initial diagnosis in our patient; however, no samples from that period are unfortunately available.

Regarding rapid immunoassays, including both chemiluminescence techniques and those using latex via immunoturbidimetric methods or gel particles, these tests generally exhibit excellent sensitivity for detecting HIT antibodies but have poor sensitivity for VITT antibodies [25, 43]. Thus, a serological profile showing strong positivity in ELISA combined with a negative result in rapid immunoassays, or the disappearance of an ELISA signal after the addition of heparin, should prompt consideration of a VITT diagnosis in a case suggestive of a PF4-disorder.

Finally, although false-positive serological tests for anti-PF4 antibodies due to the presence of antiphospholipid antibodies have been reported, this hypothesis appears highly unlikely in our case. The persistent strong positivity for anti-PF4 antibodies across three different ELISA kits, together with the transient and incomplete positivity of antiphospholipid antibodies, more likely related to the infectious syndrome than to true antiphospholipid syndrome, argues against this hypothesis.

Platelet activation functional assays are used to demonstrate the capacity of these antibodies to activate platelets from healthy donors in the presence of heparin and/or PF4. Unlike screening tests, which show wide variability in sensitivity depending on the technique used, all platelet activation assays are potentially highly sensitive for detecting both HIT and VITT entities [43]. SRA, which is the reference functional test for diagnosing HIT, is capable of detecting all three types of antibodies. However, studies have shown that the sensitivity of this test increases with the addition of exogenous PF4 [45, 46]. Moreover, adding heparin also enhances the sensitivity of the SRA for detecting HIT, but leads to a negative result or a marked decrease in positivity in cases of VITT [46], thus allowing for better differentiation between these two conditions. Among the many other available tests, it is worth highlighting the PF4-induced platelet activation assay (PIPA), which shows excellent sensitivity for both HIT and VITT antibodies, in contrast to the heparin-induced platelet activation assay (HIPA), which often yields negative results for VITT antibodies [43].

Concerning the case of our patient, it should be noted that the SRA was performed within the framework of a HIT diagnosis, at a time when knowledge of other PF4-related entities was minimal to nonexistent. As a result, the test was conducted without PF4 supplementation and in the presence of heparin, two factors known to significantly reduce the sensitivity of the SRA for detecting VITT [46], while having only a moderate effect on its sensitivity for spHIT [43].

Considering these findings, and the well-established positive correlation between ELISA serological test intensity and the likelihood of functional test positivity for VITT cases [47], the negative SRA result also raises questions about the possibility of a VITT-like syndrome, while not excluding the hypothesis of spHIT. This hypothesis is further supported by the existence of a biologically similar VITT-like case, in which strong anti-PF4/heparin IgG ELISA positivity was observed alongside a negative platelet activation test that became positive upon addition of PF4 [25]. It would, however, have been valuable, if plasma samples from the acute phase were still available, to perform an SRA supplemented with heparin and PF4, as this test remains the gold standard among functional assays for diagnosing PF4-related disorders.

Finally, the gradual disappearance of ELISA serological markers observed during follow-up in the post-acute phase does not help distinguish between the two diagnostic possibilities, as these antibodies are generally transient and tend to resolve spontaneously in all PF4-disorder pathologies, except in rare chronic cases, which are only now beginning to be described [8].

Although a definitive diagnosis cannot be formally established due to the considerable time elapsed since the acute phase of this case and the unavailability of samples from that for performing more specific laboratory tests (which represents a real limitation to this type of retrospective diagnostic reclassification), several elements nevertheless preferentially support a retrospective diagnosis of a VITT-like syndrome rather than spHIT.

It is also noteworthy that long-term follow-up of the patient, particularly during the COVID-19 vaccination period, showed no recurrence of anti-PF4 antibodies after administration of a dose of the ChAdOx1 nCoV-19 adenoviral vector vaccine. This raises the question of whether a history of PF4-related disorders may constitute an additional risk factor for the development of another condition when the presumed trigger appears to differ.

## 4. Conclusions

This clinical case illustrates the diagnostic complexity posed by PF4-disorders pathologies occurring in the absence of known heparin exposure and outside the context of recent vaccination. The diagnostic uncertainty between spHIT and VITT-like syndrome highlights the difficulty of reaching a precise diagnosis, particularly for cases that occurred prior to the recent recognition and characterization of these entities. These syndromes, often associated with severe clinical presentations, require heightened diagnostic and therapeutic vigilance.

**Figure 2:**
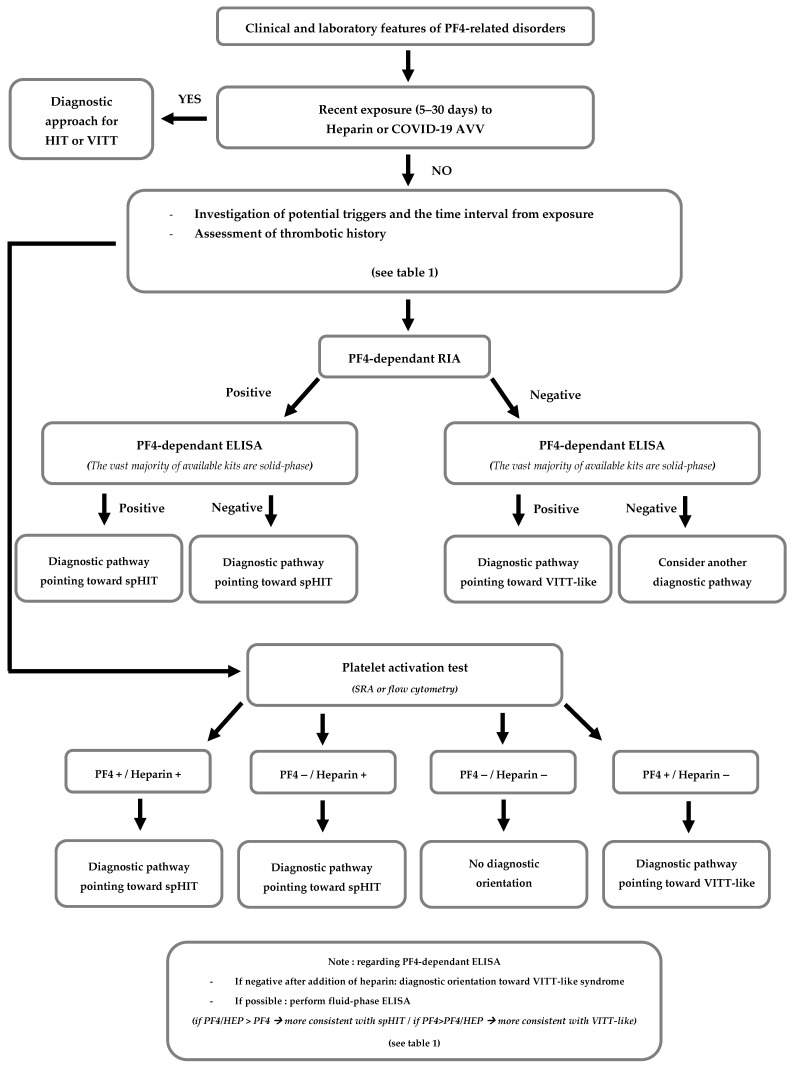
Proposed decision algorithm to guide the diagnostic orientation of PF4-related disorders. **Abbreviations:** Adenoviral vector vaccine (AAV), Rapid immunoassays (RIA).

For cases presenting a clinical and laboratory profile suggestive of PF4-related disorders, without recent heparin exposure or adenoviral vector vaccination, it is essential to identify any potential trigger and the interval between exposure and syndrome onset. Serological screening for anti-PF4 IgG antibodies performed at the time of the case should be reviewed, and combining rapid immunoassays with ELISA results can support diagnostic evaluation. Careful consideration should also be given to any functional platelet activation tests, particularly regarding positivity with or without added PF4 or heparin. Figure 2 presents a preliminary decision-making algorithm to guide the diagnostic assessment of historical PF4-disorder cases based on current knowledge.

Moreover, as highlighted by several authors of similar cases who also faced this diagnostic challenge [41], laboratory tests specifically designed for the diagnosis of VITT and VITT-like syndromes are essential to aid in the accurate identification of PF4-related disorders. In the event of a positive result, and provided that plasma samples from the time of diagnosis are available, more specific follow-up tests for each entity are ideally recommended such as fluid-phase ELISA, functional platelet activation tests with and without heparin and, if available, epitope mapping [30, 37], although these are often limited to highly specialized laboratories. While distinguishing between these entities can be subtle, it is critical both for guiding treatment decisions (non-heparin anticoagulants, intravenous immunoglobulins, immunosuppressive therapies) and for long-term management considerations (e.g., precautions during orthopedic surgery in patients with an history of spHIT or adenoviral vector vaccination in those with prior VITT-like syndrome), as well as for improving understanding of the underlying pathophysiological mechanisms and enhancing epidemiological knowledge.

This case also underscores the importance of re-evaluating past cases in light of current knowledge, and calls for the standardization of diagnostic algorithms and therapeutic strategies for these emerging PF4-dependent syndromes. The establishment of clinical registries and updated guidelines could significantly improve the prognosis of these rare, severe, and diagnostically challenging conditions.

## Data Availability

The data supporting the findings of this study are available from the corresponding author upon reasonable request. Due to patient confidentiality, some data may be restricted.

## Abbreviations

The following abbreviations are used in this manuscript:

aHIT	Autoimmune Heparin-Induced Thrombocytopenia
cHIT	Classical Heparin-Induced Thrombocytopenia
CMV	Cytomegalovirus
CRP	C-Reactive Protein
CVST	Cerebral Venous Sinus Thrombosis
ELISA	Enzyme-linked immunosorbent assay
HIPA	Heparin-induced Platelet Activation Assay
HIT	Heparin-induced Thrombocytopenia
MRI	Magnetic Resonance Imaging
OD	Optical Density
PF4	Platelet Factor-4
PIPA	PF4-Induced Platelet Activation Assay
RSV	Respiratory Syncytial Virus
SpHIT	Spontaneous Heparin-Induced Thrombocytopenia
SRA	Serotonin-release assay
UFH	Unfractionated Heparin
VITT	Vaccine-induced Thrombocytopenia and Thrombosis
